# A simulation study of the effect of lung cancer screening in China, Japan, Singapore, and South Korea

**DOI:** 10.1371/journal.pone.0220610

**Published:** 2019-07-30

**Authors:** Yufan Chen, Tina R. Watson, Steven D. Criss, Andrew Eckel, Lauren Palazzo, Deirdre F. Sheehan, Chung Yin Kong

**Affiliations:** 1 Institute for Technology Assessment, Massachusetts General Hospital, Boston, Massachusetts, United States of America; 2 Harvard Medical School, Boston, Massachusetts, United States of America; Medical University of South Carolina, UNITED STATES

## Abstract

More than 50% of the world’s lung cancer cases occur in Asia and more than 20% of cancer deaths in Asia are attributable to lung cancer. The U.S. National Lung Screening Trial has shown that lung cancer screening with computed tomography (CT) can reduce lung cancer deaths. Using the Lung Cancer Policy Model–Asia (LCPM-Asia), we estimated the potential mortality reduction achievable through the implementation of CT-based lung cancer screening in China, Japan, Singapore, and South Korea. The LCPM-Asia was calibrated to the smoking prevalence of each of the aforementioned countries based on published national surveys and to lung cancer mortality rates from the World Health Organization. The calibrated LCPM-Asia was then used to simulate lung cancer deaths under screening and no-screening scenarios for the four countries. Using screening eligibility criteria recommended by the U.S. Centers for Medicare & Medicaid Services (CMS), which are based on age and smoking history, we estimated the lung cancer mortality reduction from screening through year 2040. By 2040, lung cancer screening would result in 91,362 life-years gained and 4.74% mortality reduction in South Korea; 290,325 life-years gained and 4.33% mortality reduction in Japan; 3,014,215 life-years gained and 4.22% mortality reduction in China; and 8,118 life-years gained and 3.76% mortality reduction in Singapore. As for mortality reduction by smoker type, current smokers would have the greatest mortality reduction in each country, ranging from 5.56% in Japan to 6.86% in Singapore. Among the four countries, lung cancer screening under CMS eligibility criteria was most effective in South Korea and least effective in Singapore. Singapore’s low smoking prevalence and South Korea’s aging population and higher smoking prevalence may partially explain the discrepancy in effectiveness. CT screening was shown to be promising as a means of reducing lung cancer mortality in the four countries.

## Introduction

Lung cancer claims more lives than any other type of cancer, having accounted for 11.6% of incident cancer cases and 18.4% of cancer deaths worldwide in 2018. Over half of these incidences and disease-specific mortalities occurred in Asia.[1] Within the region, the burden of lung cancer is mostly carried by East Asia, where the 2018 age-adjusted incidence rates for men and women were higher than the global average at 47.2 and 21.9 per 100,000, respectively.[2] These figures are largely explained by tobacco use in China, where 40% of the world’s cigarettes are consumed[3].

Two other East Asian nations, Japan and South Korea, report similarly high smoking prevalence and lung cancer incidence,[4, 5] despite having “very high” human development indices (HDI), a marker of socioeconomic development.[6] In both nations, more adult males smoke on average than in other high-HDI countries.[3] As a point of comparison, Singapore, in Southeast Asia, had fewer male smokers in 2015 on average than in other high-HDI countries. Singapore is a global leader in tobacco control policy, helping to explain its comparatively low smoking prevalence.[7] Given that lung cancer is still the second most common and the deadliest cancer in Singapore, however, the disease remains a public health problem.

In 2011, the U.S. National Lung Screening Trial (NLST) found that screening with low-dose computed tomography (LDCT) in current and former heavy smokers reduced lung cancer mortality by 20% when compared to chest radiography.[8] In 2015, the U.S. Centers for Medicare & Medicaid Services (CMS) recommended that all current and former smokers ages 55 to 77 with a smoking history of at least 30 pack-years and no more than 15 years since quitting be screened for lung cancer with LDCT annually.[9] Recent findings from the Dutch-Belgian Randomized Lung Cancer Screening Trial (NELSON) study, another randomized, large-scale trial, demonstrate even greater lung cancer mortality reduction than the NLST trial, based on screening administered throughout 10 years to high-risk individuals in the Netherlands and Belgium [10].

China currently stands out among Asian countries for its progress in lung cancer screening research and policy. A notable 2010 study, for instance, investigated the feasibility of conducting population-based screening nationally.[11] In 2012, the Ministry of Health implemented the Cancer Screening Project in Urban Areas of China, which provided free LDCT screening to approximately 210,000 individuals between 2012 and 2017.[12] Amid encouraging evidence for screening outcomes and practicality, the Chinese National Health and Family Planning Commission revised its eligibility criteria for LDCT screening in 2015, capturing a subset of younger, lighter smokers compared to those accounted for in the CMS criteria.[13] The government of South Korea released lung cancer screening guidelines in the same year,[14] though officials in Japan and Singapore have yet to do so. Studies on the effectiveness and feasibility of nationwide LDCT screening in South Korea and Japan are only in their infancy, while none are currently underway in Singapore. It is necessary for screening research and policy to assume greater priority in Asia as lung cancer continues to disproportionately burden this region.

China, Japan, South Korea, and Singapore all have the resources necessary to implement national lung cancer screening programs and, furthermore, have a clear incentive to do so from a public health perspective. The purpose of our study was to use the Lung Cancer Policy Model (LCPM), a computer-based model, to simulate full implementation of national lung cancer screening in each of the four countries and estimate the resulting health benefits. We inputted high-quality data from established national surveys on smoking behavior in each country,[4, 15–17] as well as cancer incidence and mortality data collected at either the national or regional level.[2] Using eligibility criteria based on CMS guidelines, we estimated lung cancer mortality reduction due to screening from 2020 to 2040.

## Materials and methods

### The lung cancer policy model and input parameters

Proposed by American econometrician Guy Orcutt in the late 1950s,[18] microsimulation modeling has become a useful tool in health policy decision-making.[19] One significant advantage of simulation modeling is that it can integrate short-term clinical trial or health data and project the long-term consequences of a variety of scenarios. The LCPM is a well-validated microsimulation model that informed the U.S. Preventive Services Task Force’s (USPSTF) official lung cancer screening recommendations. A detailed description of the LCPM is publicly accessible through the National Cancer Institute (NCI) website[20].

The model used in this study is an altered version of the original LCPM, which we refer to as the LCPM-Asia to reflect its adaptation to Asian populations. Key input parameters include smoking intensity (number of cigarettes per day), smoking initiation and cessation rates, and mortality rates from any cause other than lung cancer. Smoking initiation and cessation rates comprise smoking prevalence and were obtained from published literature. The LCPM input files have six categories of smoking intensity, also obtained from the literature: 1–5 cigarettes per day (CPD), 6–15 CPD, 16–25 CPD, 26–35 CPD, 36–45 CPD, and 45+ CPD.[21–24] These input files include the percentage of smokers in each category. The ratio of mortality rates from any cause other than lung cancer in the four Asian countries compared to US were calculated using Global Burden of Disease data;[25, 26] the ratio was then used to scale the other-cause mortality input from the U.S. to the input from each of the four Asian countries. Additional information on model input parameters is provided in Table 1.

**Table 1:** Lung Cancer Policy Model–Asia input parameters and calibration targets.

			China	Japan	Singapore	South Korea
**Input parameters**	**Smoking cessation rate**	**Values**	Male-Age 30±65: 0.02; Age >65: 0.03 / Female-Age >35: 0.02	The same as China	The same as China	The same as China
		**Source**	Levy et al 2014			
	**Cigarettes per day**	**Values**	Male by age in 2006 (Chen, average of urban and rural)—Age 37: 16.56; Age 46: 17.44; Age 55: 15.76; Age 66: 13.2; Age 74: 12.2; Male and female by year (Ng)—1980: 15.5 (13.9, 17.1); 1996: 18.2 (17.3, 19.1); 2006: 21.8 (20.6, 23.0); 2012: 22.3 (20.7, 24.4); Male by year (Qian, calculated)—1998: 15.8; 2003: 21.3; Female by year (Qian, calculated)—1998: 12.3; 2003: 15.8	In 2007—Male: <10 19.1%, 10–19 48.6%, >20 32.3%; Female: <10 34.4%, 10–19 52.8%, >20 12.8%	In 2004—Male: <10 35%, 10–19 48%, >20 17%; Female: <10 67%, 10–19 26%, >20 7%	In 2010—Male: <10 34.4%, 10–19 52.8%, >20 12.8%; Female: <10 41.1%, 10–19 49.3%, >20 9.5%
		**Source**	Chen et al 2015; Ng et al 2015; Qian et al 2010	Tabuchi et al 2016	National Health Survey	Korea National Health and Nutrition Examination Survey (KNHANES)
	**Other-cause mortality**	**Values**	Individualized based on age and sex	Individualized based on age and sex	Individualized based on age and sex	Individualized based on age and sex
		**Source**	Global Burden of Disease; 1990 and 2010	Global Burden of Disease, 1990 and 2010	Global Burden of Disease, 1990 and 2010	Global Burden of Disease, 1990 and 2010
**Calibration targets**	**Smoking prevalence**	**Values (in 2010)**	Male: 44.8% / Female: 2%	Male: 26.6% / Female: 9.3%	Male: 17.9% / Female: 6.3%	Male: 33.5% / Female: 8.8%
		**Source**	China Health and Nutrition Survey	JT's Annual Japan Smoking Rate Survey; National Health and Nutrition Survey	National Health Survey; National Health Surveillance Survey	Korea National Health and Nutrition Examination Survey (KNHANES)
	**Lung cancer mortality**	**Values**	Male 422,000 / Female: 175,000	Male: 53,976 / Female: 21,367	Male: 1,083 / Female: 507	Male: 12,783% / Female: 5,065
		**Source**	GLOBOCAN^a^ 2012	GLOBOCAN^a^ 2012	GLOBOCAN^a^ 2012	GLOBOCAN^a^ 2012

Given the insufficient information on smoking cessation rates in Japan, South Korea and Singapore, we applied the smoking cessation rate in China to the other countries.

^a^ GLOBOCAN: Datasets about the global burden of cancer released by the International Agency for Research on Cancer (IARC)

The calibration targets were smoking prevalence and lung cancer mortality. The model outputs are calibrated to targets derived from the literature. The four Asian countries we studied all publish national health survey results every one to three years; these results provided us with each country’s smoking prevalence by gender. Lung cancer mortality rates were derived from the International Agency for Research on Cancer (IARC) GLOBOCAN project, extracted from the World Health Organization database from 1987 to 2012.[27] Additional information on calibration targets is provided in Table 1.

For each run of the model in each of the four countries, the LCPM-Asia simulated a population of individuals using a multiple birth cohort approach.[28] Modeling multiple birth cohorts provides a more accurate estimation of health benefits than would the use of a single birth cohort, as smoking behavior and lung cancer risk vary drastically with age.[28] Our methods therefore provide robust estimations of the true benefits of screening to the study populations. All simulation outputs were scaled to estimates for actual national populations from the U.S. Census Bureau International Data Base, which projects population by age[29].

### Screening strategies

The calibrated model simulated two screening scenarios within each of the four countries and estimated the resulting health effects. No lung cancer screening was the base case scenario. Lung cancer screening according to CMS guidelines was the alternative. In this latter scenario, current and former smokers ages 55–77 with at least 30 pack-years of smoking history and fewer than 15 years since quitting were screened for lung cancer annually with LDCT, beginning in 2020 and ending in 2040. We assumed 100% screening adherence on a nation-wide level to assess the full potential harms and benefits of screening. The effects of smoking cessation rate and smoking intensity are addressed in the sensitivity analysis.

### Simulation outcomes

The simulation generated the number of lung cancer deaths within each smoking category—current, former, and never smokers—each year from 2020 to 2040. For each country, annual lung cancer mortality reduction was calculated by subtracting the number of lung cancer deaths in the screening scenario from the number of lung cancer deaths in the no-screening scenario in the same year, then dividing by the latter (the baseline mortality). Cumulative lung cancer mortality reduction was derived from these annual mortality reductions.

The percentage of the population eligible for screening each year was calculated by dividing the total number of LDCT screening exams that year by the total adult population. The cumulative life-years saved from 2020 to 2040 was calculated by subtracting each country’s cumulative estimated population during 2020 to 2040 in the no screening scenario from the cumulative estimated population during these years in the screening scenario. Life-years saved per lung cancer death prevented was calculated by dividing life-years saved by the cumulative number of lung cancer deaths prevented.

### Sensitivity analysis

Sensitivity analyses were conducted to anticipate variability in smoking behaviors in a realistic situation. Following Jeon et al.’s method,[30] we simulated an optimistic and a pessimistic scenario. In the optimistic alternative, smoking cessation rates were 20% higher and mean cigarettes per day, a measure of smoking intensity, were 20% lower for birth cohorts after 1981 starting in the year 2011. In the pessimistic scenario, smoking cessation rates were 20% lower and smoking intensity was 20% higher beginning with the same year and birth cohort.

## Results

### Model calibration

**Fig 1:**
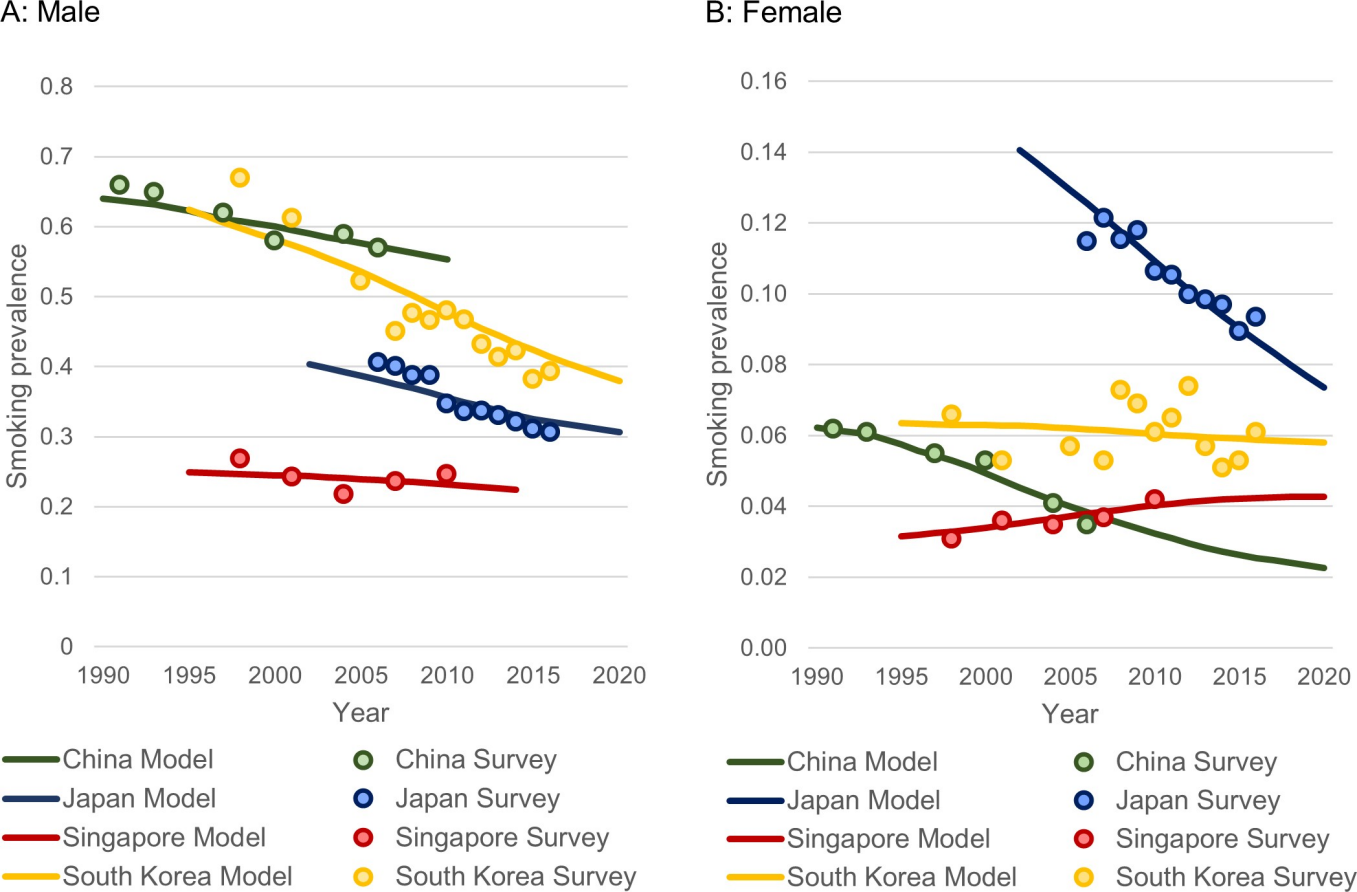
Smoking prevalence calibration. Model smoking prevalence (lines) calibrated to actual data from each country’s survey (dots). (A) Male. (B) Female.

**Fig 2:**
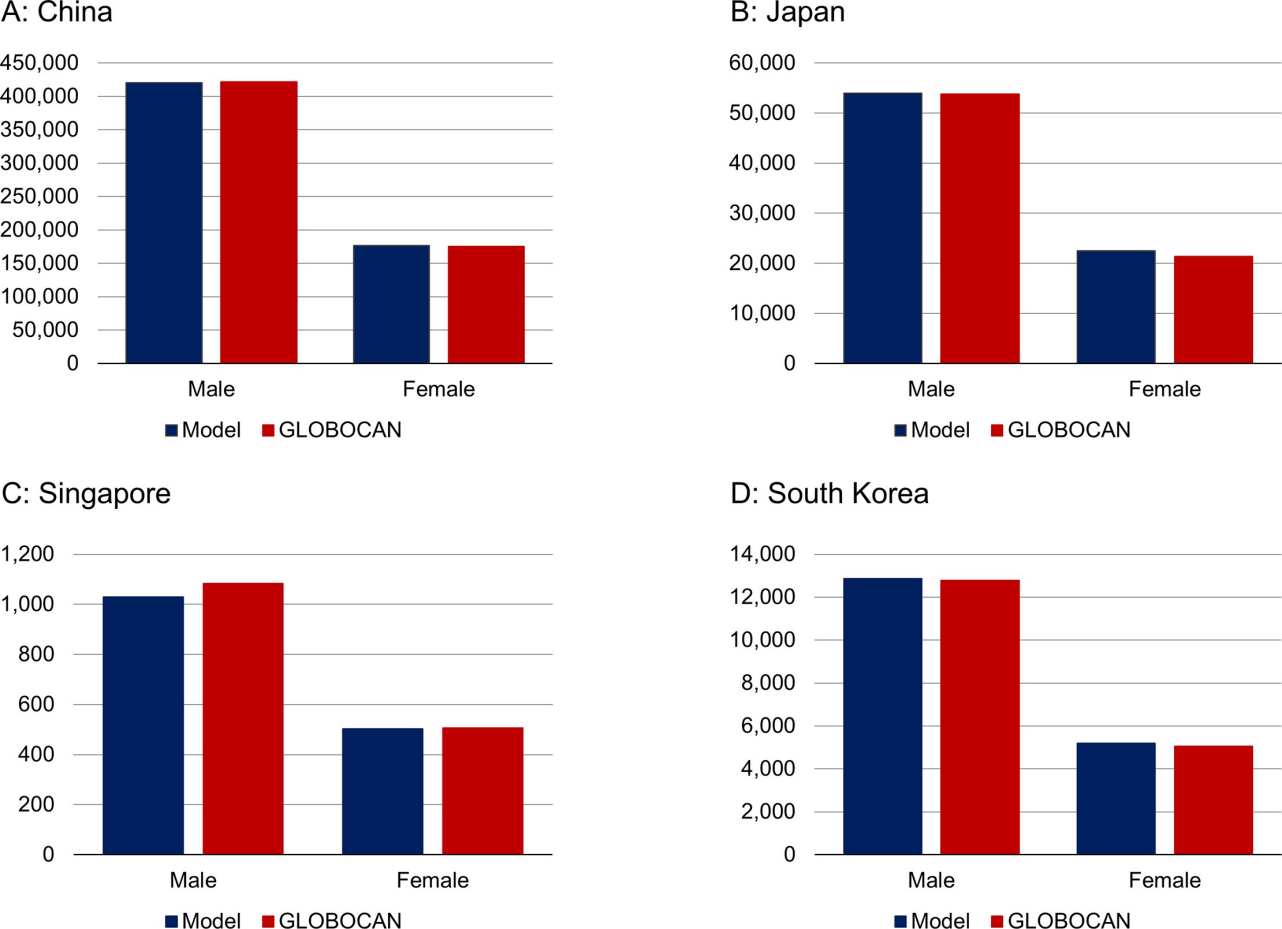
Lung cancer mortality calibration. Model lung cancer mortality calibrated to data from GLOBOCAN. GLOBOCAN: Datasets about the global burden of cancer released by the International Agency for Research on Cancer (IARC). (A) China. (B) Japan. (C) Singapore. (D) South Korea.

Smoking prevalence outputted by the LCPM-Asia was calibrated to data from national surveys. We calibrated male and female smoking prevalence separately by country (Fig 1). Lung cancer mortality calibration results are shown in Fig 2.

### Eligibility for screening

**Fig 3:**
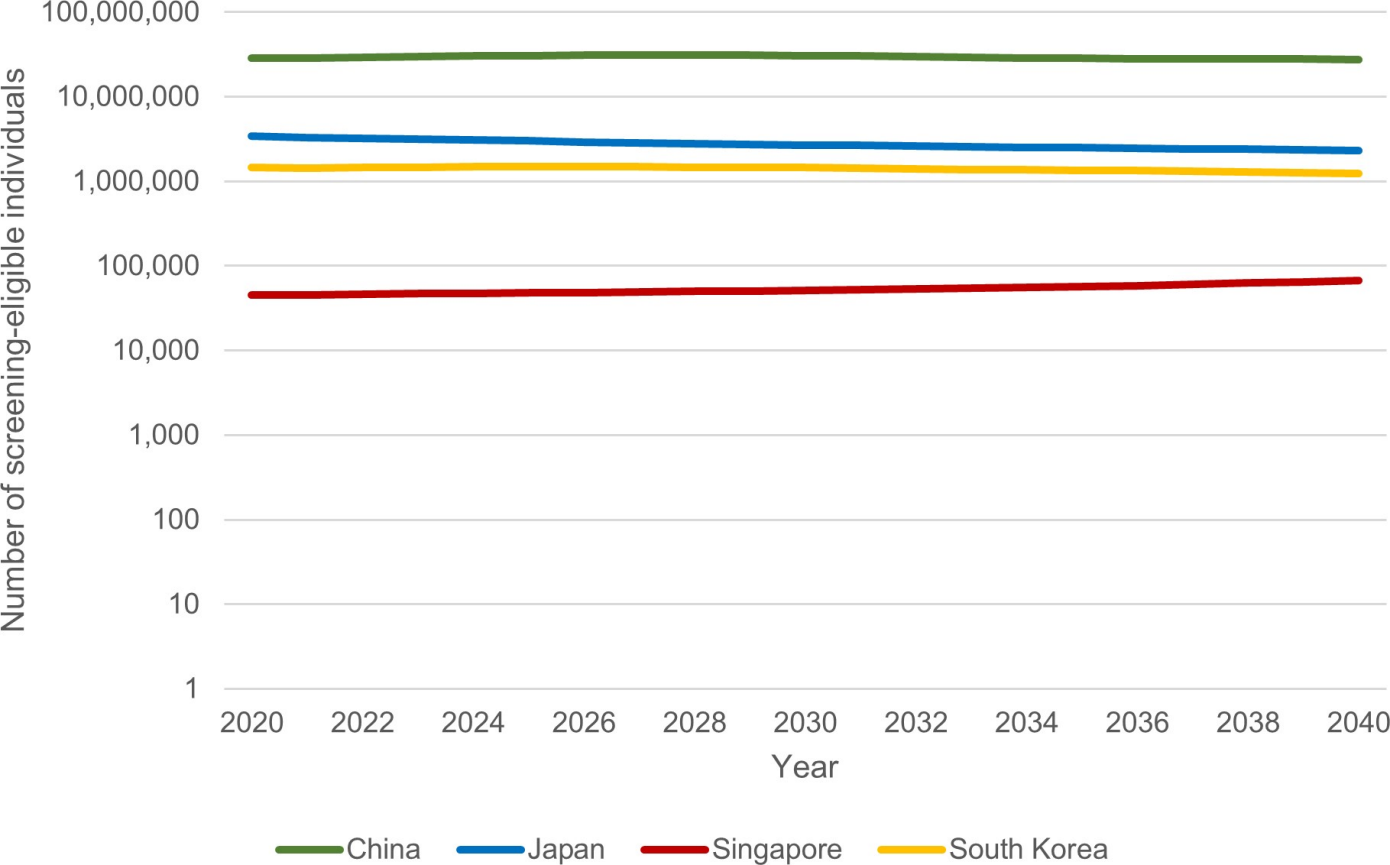
Number of people eligible for screening, 2020–2040. Y-axis uses a logarithmic scale.

**Fig 4:**
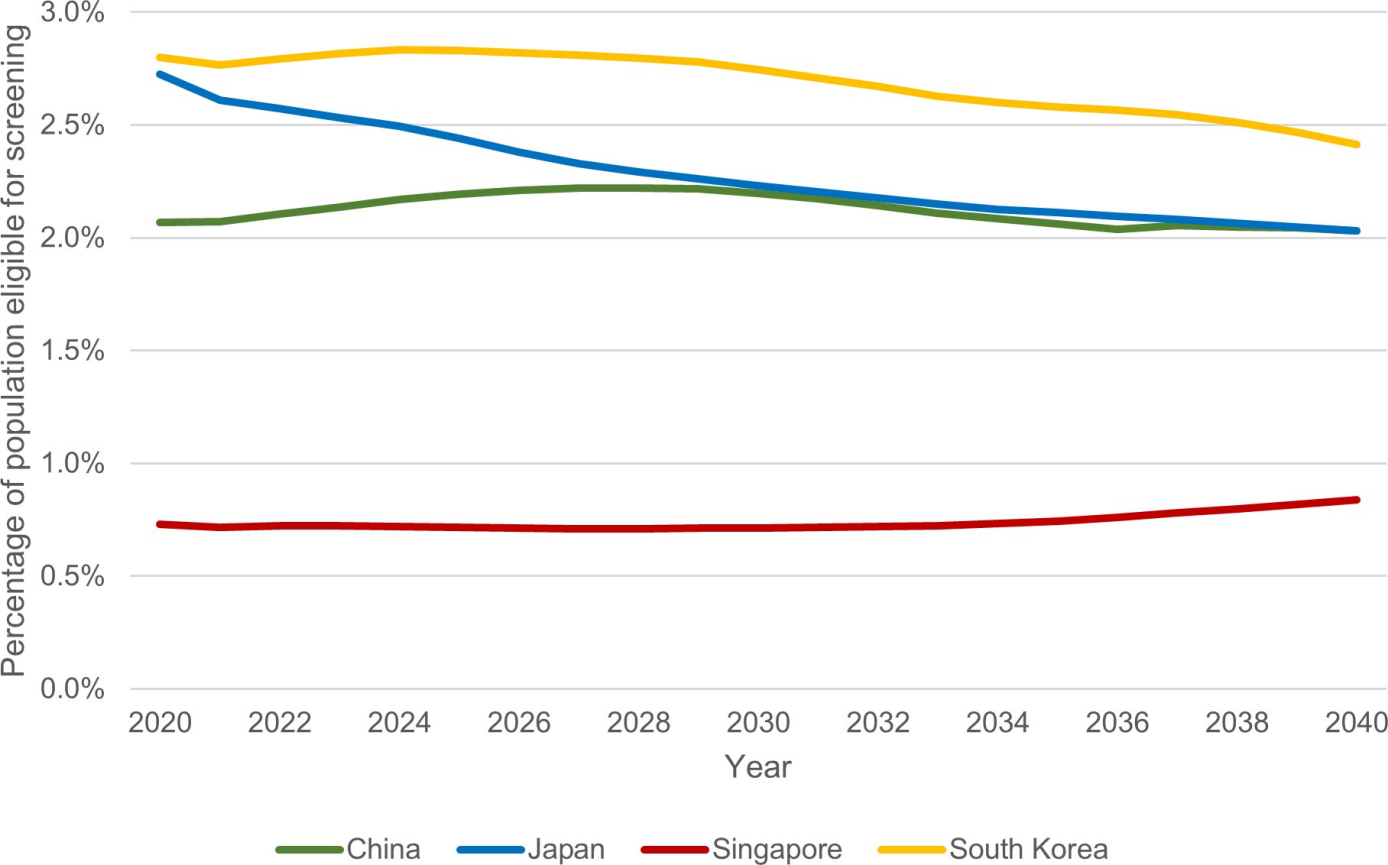
Percentage of population eligible for screening, 2020–2040.

We estimated the number of screening-eligible individuals in each country if CMS guidelines were implemented. Estimates for each year, from 2020 to 2040, are shown in Table 2. Throughout the study period, China had the highest estimated number of screening-eligible individuals, equivalent to the number of annual screens calculated by the model—about 30,000,000. In the remaining countries, the annual number of screens ranged from about 50,000 in Singapore to around 3,000,000 in Japan for any year between 2020 and 2040. Trends in the number of screens per country from 2020 to 2040 can be found in Fig 3. The estimated percentage of each country’s total population that would be eligible for screening, by year, is shown in Fig 4.

**Table 2:** Projected population and number of screening-eligible individuals, 2020–2040.

		2020	2030	2040
**China**	**Population**	1,384,545,220	1,391,490,898	1,358,518,748
	**Screens**	28,622,658	30,570,342	27,603,631
**Japan**	**Population**	125,507,472	120,751,317	114,448,328
	**Screens**	3,419,291	2,694,940	2,322,707
**Singapore**	**Population**	6,209,660	7,222,632	8,035,916
	**Screens**	45,394	51,587	67,271
**South Korea**	**Population**	51,835,110	52,792,497	51,328,829
	**Screens**	1,451,977	1,449,188	1,238,713

### Mortality reduction

Using the LCPM-Asia, we estimated that, by 2040, lung cancer screening would result in cumulative mortality reductions ranging from 3.76% to 4.74% between the four countries (Table 3). South Korea derived the greatest benefit in this regard (4.74% cumulative mortality reduction), followed by Japan (4.33%), China (4.22%), and Singapore (3.76%). The greatest number of projected deaths avoided, however, were in China (471,095), the country with the largest population. We estimated that 45,774 deaths were avoided in Japan, followed by 14,504 in South Korea and, lastly, 1,290 in Singapore. In each country, cumulative mortality reduction among current smokers, ranging from 5.56% in Japan to 6.86% in Singapore, was higher than overall mortality reduction and mortality reduction among former smokers. Mortality reduction among former smokers ranged from 3.69% in Japan to 4.68% in South Korea.

**Table 3:** Projected lung cancer morality reduction and deaths avoided by smoker type, 2020–2040.

	Total		Current smoker		Former smoker	
	Mortality reduction	Deaths avoided	Mortality reduction	Deaths avoided	Mortality reduction	Deaths avoided
**China**	4.22%	471,095	5.98%	328,959	4.07%	142,137
**Japan**	4.33%	45,774	5.56%	32,066	3.69%	13,708
**Singapore**	3.76%	1,290	6.86%	909	4.62%	381
**South Korea**	4.74%	14,504	6.75%	10,089	4.68%	4,415

**Fig 5:**
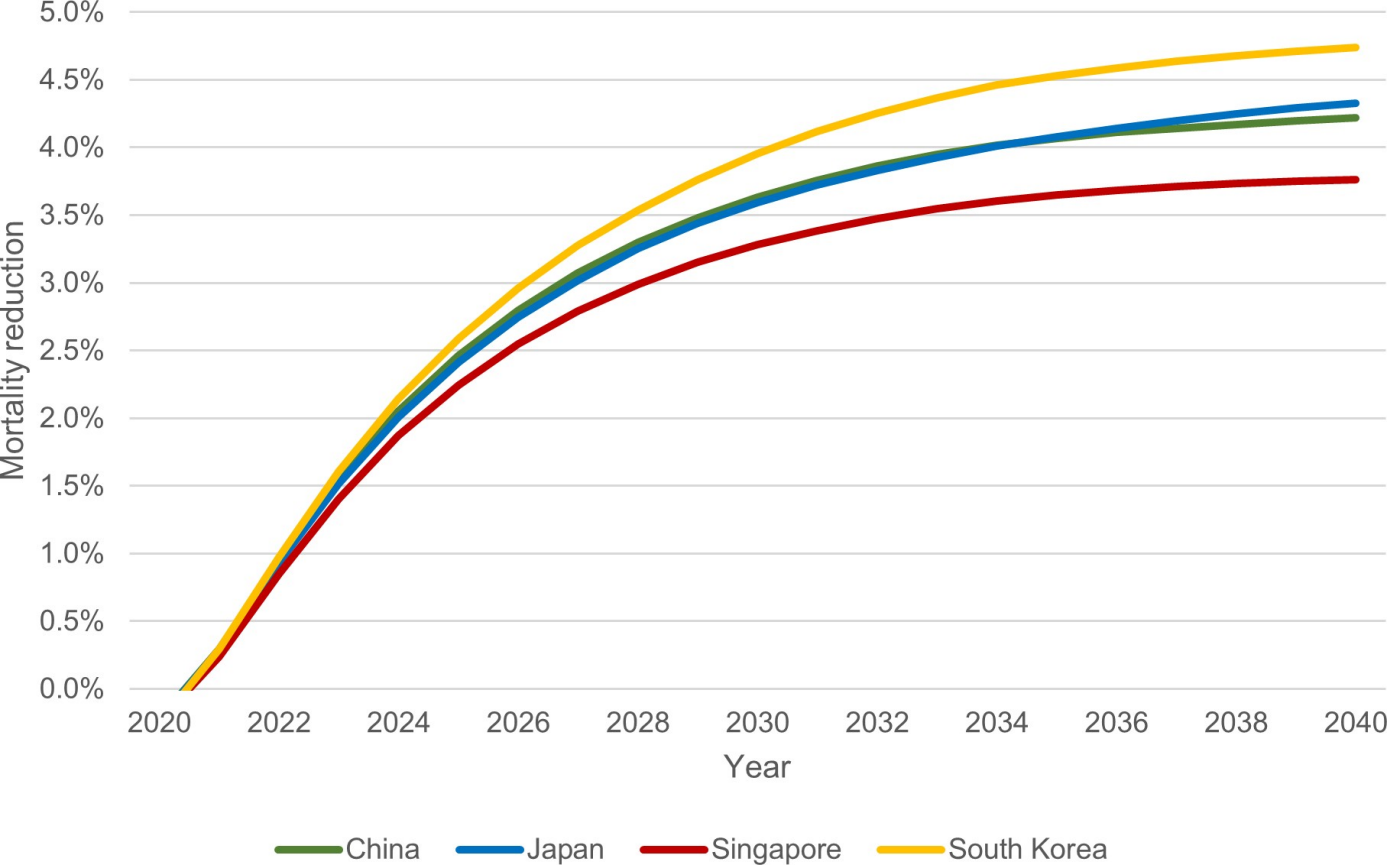
Cumulative mortality reduction by year, 2020–2040.

Cumulative mortality reduction trends from 2020 to 2040 are shown in Fig 5. Trends were projected to reach a plateau around the year 2036. At any point in time, the highest and lowest cumulative mortality reductions were estimated to be in South Korea and Singapore, respectively. Throughout the study period, cumulative mortality reductions in China and Japan were similar.

**Fig 6:**
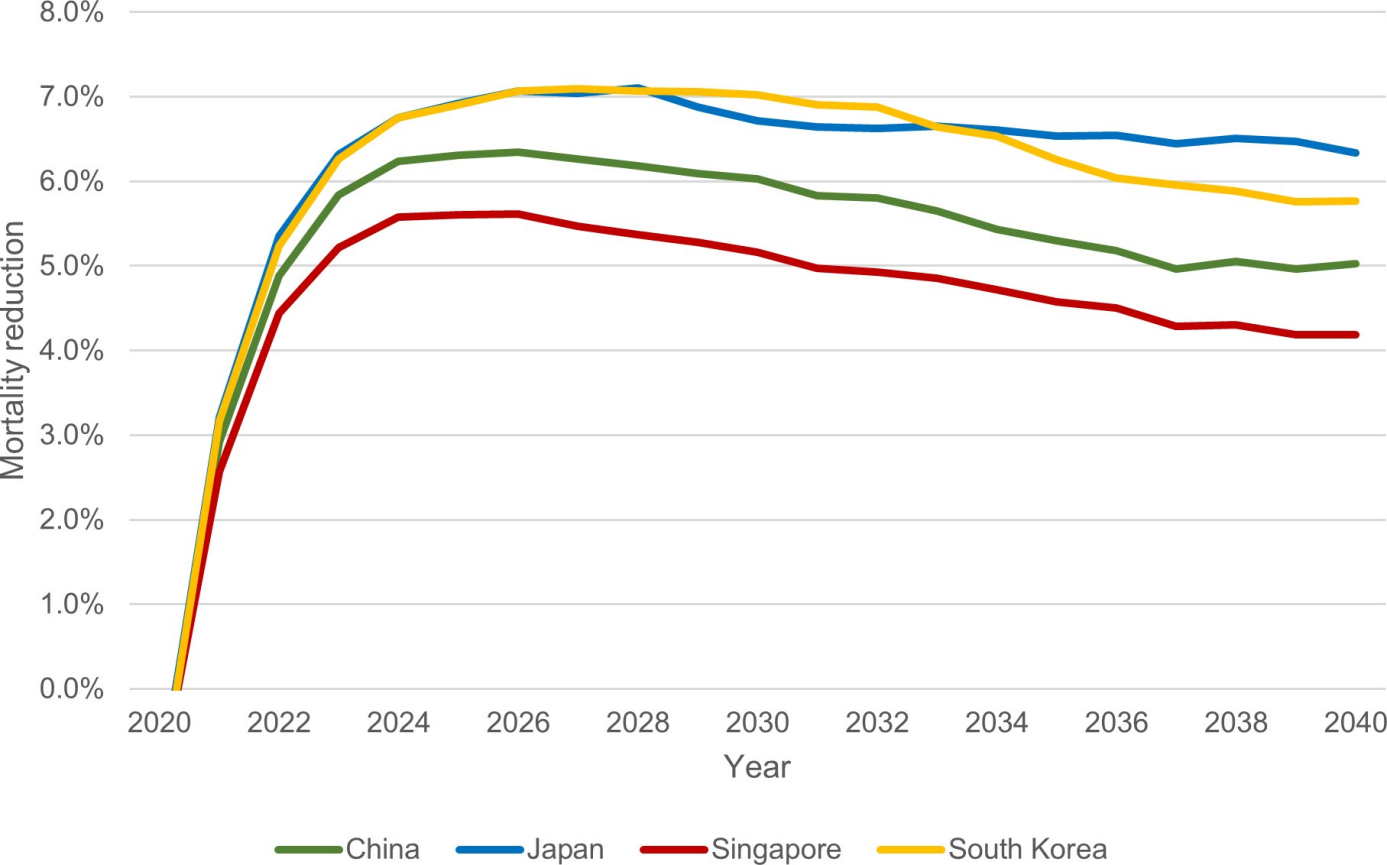
Annual mortality reduction by year, 2020–2040.

Total annual mortality reductions from 2020 to 2040 for each country are presented in Fig 6. In all countries, mortality reduction peaks in the year 2026 after a steep increase during the six years immediately following implementation of screening, and then begins to steadily decrease before flattening.

### Life-years gained

As shown in Table 4, full implementation of lung cancer screening from 2020 to 2040 was estimated to save the greatest number of life-years in China (3,014,215) and the least in Singapore (8,118). Greater population size corresponds to a greater number of individual lives prolonged by early lung cancer detection and, by extension, more life-years gained in the screening scenario. However, we estimated that the number of life-years saved per lung cancer death prevented remained relatively constant between countries.

**Table 4:** Projected life-years gained, 2020–2040.

	China	Japan	Singapore	South Korea
**Life-years gained**	3,014,215	290,325	8,118	91,362
**Lung cancer deaths prevented**	471,095	45,774	1,290	14,504
**Life-years saved per lung cancer death prevented**	6.40	6.34	6.29	6.30

### Sensitivity analysis

Sensitivity analyses estimated the survival benefit of lung cancer screening if smoking intensity and cessation rates were different from those values originally inputted into the model. The purpose of these analyses was to anticipate the variability in future smoking behaviors. The consequent changes in mortality reduction over time are shown in Table 5. In 2040, cumulative mortality reductions in the optimistic, original, and pessimistic scenarios are nearly equivalent within each country. In China, for example, lung cancer mortality reduction was 4.215% and 4.219% in the optimistic and original scenarios, respectively; the number of deaths avoided were, respectively, 470,542 and 471,095.

**Table 5:** Sensitivity analysis: Mortality reduction through 2040 with varying smoking intensity and cessation rate.

		Original scenario	Optimistic scenario	Pessimistic scenario
**China**	**Mortality reduction**	4.219%	4.215%	4.223%
	**Deaths avoided**	471,095	470,542	471,727
**Japan**	**Mortality reduction**	4.329%	4.327%	4.333%
	**Deaths avoided**	45,774	45,743	45,823
**Singapore**	**Mortality reduction**	3.765%	3.757%	3.775%
	**Deaths avoided**	1,290	1,287	1,294
**South Korea**	**Mortality reduction**	4.739%	4.738%	4.750%
	**Deaths avoided**	14,504	15,501	15,549

In the optimistic alternative, smoking cessation rates were 20% higher and mean cigarettes per day, or smoking intensity, 20% lower for future birth cohorts after 1981 from the year 2011. In the pessimistic scenario, smoking cessation rates were 20% lower and smoking intensity 20% higher beginning with the same year and birth cohort

## Discussion

Using the LCPM-Asia, we estimated that lung cancer screening through 2040 would result in cumulative mortality reductions ranging from 3.76% to 4.74% among the four countries we studied. Life-years saved ranged from 8,118 in Singapore to 3,014,215 in China. With the highest number of life-years saved, China would also carry the greatest burden of screening, with about 30 million screens performed per year. In contrast, about 50,000 screens per year were estimated to take place in Singapore.

Lung cancer screening had the least projected influence on cumulative mortality reduction in Singapore, amounting to 3.76% in a twenty-year period. Singapore’s strict tobacco control policies result in low smoking prevalence as compared to the other three Asian countries.[31, 32] In consequence, the percentage of the projected population eligible for screening is consistently lowest in Singapore, and the overall population derives less of a benefit from early detection of cancer. Screening in South Korea yields the highest mortality reduction at 4.86%. It may be expected that China would benefit the most from screening in this regard, given the high smoking prevalence among Chinese men. However, the low percentage of Chinese women who smoke (about 2%) has the effect of lowering the projected cumulative mortality reduction in China. With its large smoking and aging population, South Korea has more to gain from the implementation of screening, as a larger portion of its population would be screening-eligible. While Japan has a comparatively large aging population due to improved living standards and medical care over the past few decades,[33] a lower mortality reduction is observed compared to South Korea, possibly due to the lower smoking prevalence among Japanese men.

After altering smoking cessation rates and smoking intensity by 20% in a sensitivity analysis, no substantive differences in lung cancer mortality were observed. This may be due to the fact that the impacts of tobacco control are delayed, given that lung cancer risk only begins to decline years after the actual cessation of smoking or diminishing of smoking intensity.[34, 35] The ultimate benefit of tobacco control programs, however, is clear—smoking prevalence and lung cancer mortality in many developed countries, such as Australia [36], the U.S. [37], and Hong Kong [38], have declined as a result of such measures. Although China joined the WHO Framework Convention on Tobacco Control (FCTC) in 2003, implementing tobacco control is especially difficult in China since the China National Tobacco Corporation, which holds a monopoly over the industry, is a state-owned enterprise.[39] Meanwhile, as was previously mentioned, Chinese health officials have made progress in confronting these limitations by releasing official guidelines for LDCT screening.[13] If implemented in China with full adherence, these screening guidelines would prevent about 20,000 (2.9%) more lung cancer deaths in China through 2050 than the CMS guidelines would, but at a price of about 445 million (44.7%) more screens.[40] A combination of tobacco control programs and a lung cancer screening guideline fitted to the population would likely be most effective in China, as is corroborated by our previous study reporting a greater mortality reduction rate if smoking cessation interventions were added to an annual lung cancer screening program in the United States.[34]

Although official LDCT lung cancer screening guidelines have yet to be released in South Korea and Japan, studies have explored the possibility of implementing screening programs in these countries. Using eligibility criteria suggested by South Korean government officials,[14] a pilot study on the feasibility of organizing nationwide lung cancer screening was conducted in South Korea from 2016 to 2017, with similar or less favorable results than reported in previous studies on LDCT.[41] However, a national screening program for other prevalent cancers in South Korea has reported encouraging adherence rates and outcomes,[42, 43] indicating that a program targeting lung cancer may be similarly successful, especially given the current consensus among Korean lung cancer specialists on the net benefit of screening.[44] In Japan, nationwide screening programs with chest radiography have been traditionally conducted. However, according to the results from the Prostate, Lung, Colorectal, and Ovarian (PLCO) Cancer Screening Trial, chest radiography screening was not effective in reducing the lung cancer mortality rate.[45] Several case-control studies in Japan also showed that the benefit from chest radiography screening was limited and suggested the need for a more powerful screening modality.[46] A large-scale study conducted in Hitachi City from 1998 to 2012 comparing LDCT and chest radiography screening concluded that LDCT may be promising as an early detection method for lung cancer.[47–49] Although numerous studies have indicated that LDCT screening would be beneficial in South Korea and Japan, no simulation studies have, as of yet, investigated the effectiveness of LDCT screening in the future. The results of our study may, therefore, be used to assist Korean and Japanese policy makers in evaluating the benefits of lung cancer screening.

Singapore stands out in our analysis for having the lowest overall mortality reduction, number of life-years gained, and percentage of the population eligible for screening, seeming to benefit the least from LDCT. Indeed, the most recent statement on the subject from Singapore’s Ministry of Health recommended in 2010 that LDCT not be used to scan for lung cancer outside of a clinical trial.[50] Although screening is not favorable for the population as a whole, due to Singapore’s strict tobacco control, our analysis reveals some benefits for ever-smokers—the projected mortality reductions due to screening among current and former smokers are higher in Singapore (6.86% and 4.62%, respectively), compared to the other three countries. Taking these results into account, future efforts should be dedicated to understanding how to best structure lung cancer screening guidelines in Singapore.

### Limitations

To study cancer control in four Asian countries, we used screening guidelines that were modeled after the U.S. population. Smoking behavior and age distribution vary considerably between U.S. and Asian populations, as well as among the four Asian populations we studied. An example is the much smaller gender discrepancy in smoking prevalence in the U.S.[51] If modeled after the unique smoking behaviors and demographics of each country, screening guidelines would be optimized to provide the greatest benefit.

Second, our model is calibrated to the results of the NLST,[52–55] which could be considered relatively outdated, as this study was started in 2002. Since the NLST study period, advancements in lung cancer imaging techniques could provide even greater efficacy than was shown in the NLST. Improved screening technology could lead to a greater yield with lung cancer screening and allow for accurate screening over a longer period of time (USPSTF recommendations suggest screening until age 80, compared to 77 in the CMS guidelines). Using data from more recent lung cancer screening trials, such as NELSON, the UK Lung Cancer Screening Trial (UKLS) and the International Early Lung Cancer Action Program (I-ELCAP), might offer a solution. However, implementing the results of these trials would come with additional limitations. NELSON and UKLS have not yet openly published their data in full; furthermore, each participating I-ELCAP institution had its own eligibility criteria, limiting the utility of the study results.[56] Therefore, the NLST results are currently the most complete dataset available for evaluating the effectiveness of lung cancer screening. Future simulation studies should be performed using data from these more recent trials once complete data becomes available.

An additional limitation concerns the fact that cigarette use is not the sole cause of lung cancer. Studies have shown that the incidence of lung cancer among non-smokers in Asian countries is higher than that in Europe and the U.S.[57] Air pollution, indoor cooking oil vapor, coal burning, and environmental tobacco smoke are serious risk factors for lung cancer in Asia, particularly in China, and cause the majority of cases in non-smokers.[58, 59] The effects of these causes are not factored into our model. Furthermore, the study conducted in Hitachi City, Japan, concluded that LDCT screening for a population including non-smokers and light smokers may be effective,[49] suggesting that developing screening guidelines that include non-smokers and light smokers could be beneficial for Asian countries.[49] However, in order to fully understand the impact of inclusion of non-smokers and light smokers in the screening program, many other health outcomes that were not reported in the Hitachi City study need to be considered, such as radiation-induced cancers, overdiagnosis rates, and false-positive rates. Thus, we cannot use the Hitachi City study to inform the screening of non-smokers and light smokers.

Lastly, the condition of 100% screening adherence used in our study was intended to demonstrate the potential extent of positive impact if national lung cancer screening were implemented. However, the challenges involved in maintaining high levels of adherence, let alone full adherence, are considerable. In a realistic scenario, the benefits of screening would likely be more moderate than the estimations we present here. The extent of adherence would also be difficult to predict.

## Conclusion

Ours is the first simulation study that assesses the impact of national LDCT screening in Japan, Singapore, and South Korea. Under the assumption of full adherence to screening guidelines recommended by CMS, lung cancer mortality reduction and life-years saved were projected between the years 2020 and 2040 in these three countries and in China. LDCT screening was estimated to be most effective in South Korea and least effective in Singapore; these results reflect the smoking profiles and age distributions of these countries. To maximize the benefit of LDCT screening, each country should establish a screening guideline fitted to its own demographic makeup and smoking profile.
